# Awareness of and receptivity to FDA’s point-of-sale tobacco public education campaign

**DOI:** 10.1371/journal.pone.0288462

**Published:** 2023-07-13

**Authors:** Lauren M. Dutra, Matthew C. Farrelly, Brian Bradfield, Debra Mekos, Chaunetta Jones, Tesfa Alexander

**Affiliations:** 1 Center for Health Analytics, Media, and Policy, RTI International, Raleigh, North Carolina, United States of America; 2 Center for Tobacco Products, United States Food and Drug Administration, Silver Spring, Maryland, United States of America; 3 Public Health Systems Innovation and Transformation, MITRE Corporation, Rockville, Maryland, United States of America; Universidad de Alcala, SPAIN

## Abstract

The purpose of the study was to assess awareness of and receptivity to FDA’s point-of-sale (POS) tobacco public education campaign for adult cigarette smokers called *Every Try Counts*; it was the first multi-county POS campaign in the U.S. The design was a county-level treatment-control three-wave longitudinal design. The setting was 15 treatment and 15 control counties. Subjects were smokers ages 25 to 54 (N = 3,628). 4,145 individuals screened in as eligible; 3,628 (87.5% response rate) completed the Wave 1 questionnaire (Wave 2: n = 2,812; Wave 3: n = 2,571; retention 70.9%). Measures were self-reported brand and ad awareness (saw any ad a few times or more) and receptivity (5-item perceived effectiveness scale). The analysis included descriptive analyses of receptivity; bivariate analyses of awareness by treatment group; and covariate- and time-adjusted logistic regression models to determine changes in awareness attributable to the campaign. Receptivity was moderate and differed significantly by race/ethnicity. As was the case for all waves, at wave 3, ad awareness was significantly higher in treatment (53.3%) than control counties (36.1%, p < .05). In regression models, brand (OR = 1.53, 95% CI: 1.26–1.86) and ad (OR = 1.74, 95% CI: 1.39–2.16) awareness were significantly higher in treatment than control counties. *Every Try Counts* generated a moderate level of receptivity and attention from cigarette smokers. Limitations include self-reports of campaign awareness and generalizability to a small number of U.S. counties.

## Introduction

The tobacco industry spends most of its marketing and advertising budget at the point of sale (POS) [1], concentrating much of this spending in low-income and predominately African-American and Hispanic communities [2–4]. Retail establishments feature discounts, price promotions, and strategically placed and visually appealing advertisements and product displays. Research suggests that POS tobacco advertising serves as a barrier to smoking cessation and abstinence among adult smokers [5–8]. Exposure to POS tobacco advertising has been associated with cravings to smoke among former smokers and fewer quit attempts among current smokers [5, 6].

Mass media campaigns are an important component of tobacco education and have been shown to affect tobacco-related knowledge attitudes, and beliefs and tobacco use behavior in a variety of populations [2, 9–17]. However, to date, limited research has examined awareness and effectiveness of POS tobacco education campaigns in the U.S. [18]. The one exception is evaluation of the New York City Board of Health’s 2009 regulation, which required grocery stores and pharmacies to post graphic warning signs next to registers and tobacco product displays; the ads highlighted the negative health effects of smoking [19]. Intercept surveys with current and former smokers conducted prior to and nine months after the ads were installed found that awareness of the POS signs more than doubled over time, and thoughts about quitting increased by 11% [19].

Virtual convenience store research provides additional insights on how smokers might respond to a POS campaign [20–24]. One virtual store experiment found that exposure to supportive messaging was associated with a lower urge to smoke among adult smokers [22]. In a similar experiment, smokers and recent quitters reported feeling more motivated and hopeful after viewing ads that emphasized the benefits of quitting smoking cigarettes and more negative emotions and affective dissonance after viewing graphic ads focused on the social consequences of smoking [24].

### Description of the campaign

In January 2018, the U.S. Food and Drug Administration (FDA) launched *Every Try Counts*^™^ (*ETC)*, the first multi-county POS tobacco public education campaign in the U.S. The campaign ran for two years (2018 and 2019) in 15 counties that had a high prevalence of adult cigarette smokers and sufficient POS advertising space to meet the Center for Disease Control and Prevention’s (CDC’s) recommended minimum of 800 gross rating points (GRPs) per quarter [25] (see S1 File for the approach to measuring GRPs for this campaign and the GRP levels achieved by the campaign). The campaign targeted adult cigarette smokers ages 25–54 who wanted to quit but had been unsuccessful in the past. The goal of the campaign was to strengthen smokers’ motivation to quit by encouraging them to “practice the quit” [26]. This unique motivational approach (as opposed to the graphic advertising approach used by many previous U.S. campaigns [11, 17, 19, 27]) was based on formative research (56 focus groups and 1,500 smokers) conducted by the campaign developer, FCB New York (FCB NY). In focus groups, adult smokers expressed a preference for *ETC’s* encouraging tobacco education messages over graphic ads.

**Fig 1:**
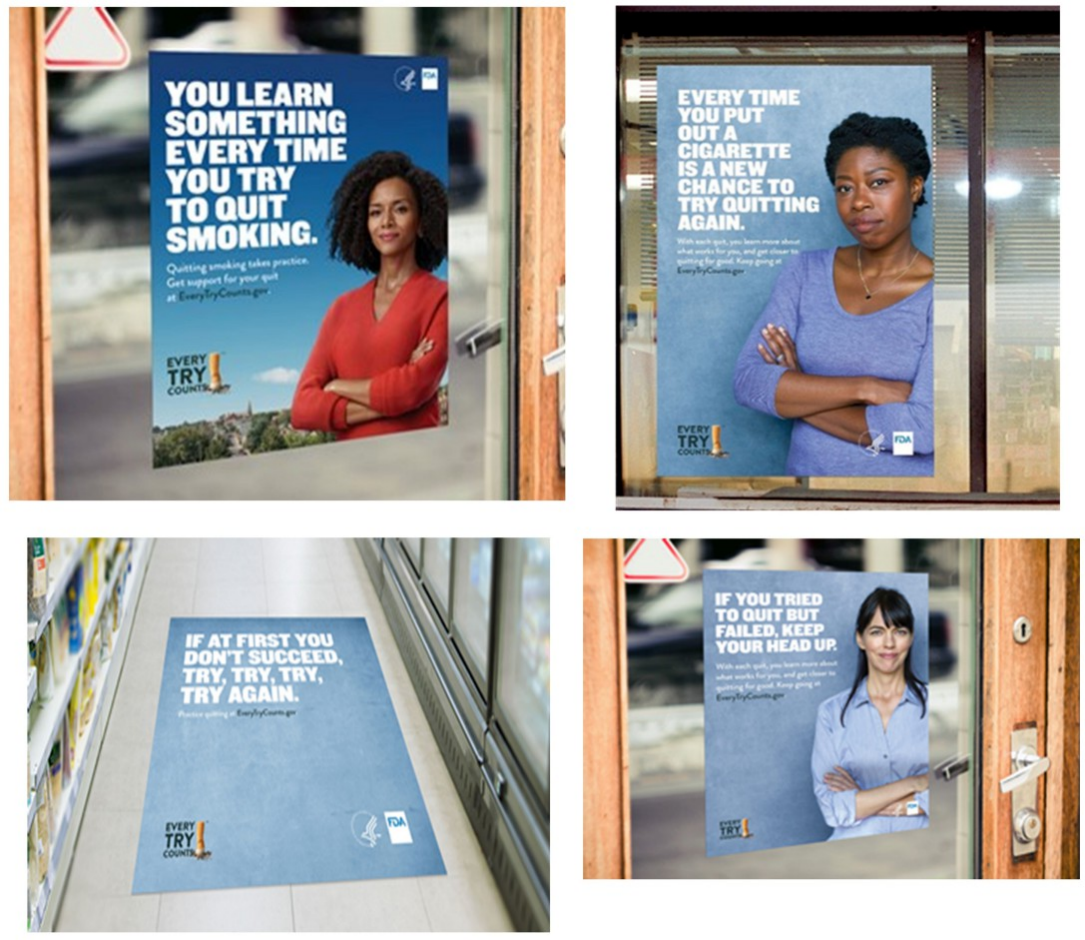
Examples of *Every Try Counts* point-of-sale print ads (in context in a convenience store environment). This figure includes photos of ads used in the campaign.

Campaign materials included posters, door and floor clings, counter mats, coffee cup sleeves, ATM and gas pump toppers, and nearby billboards (Fig 1). All ads contained the FDA and *ETC* logos and the URL for the campaign website (www.everytrycounts.gov), which emphasized the health benefits of quitting and featured cessation tools developed by the National Cancer Institute. Larger ads featured a slogan or tagline (e.g., “You didn’t fail at quitting, you just haven’t finished the process”). Store participation in the campaign was optional in treatment counties; store owners could refuse to feature the ads in their stores. However, the refusal rate for ad placement was very low. To augment the print campaign, FCB NY ran targeted digital video ads in all campaign counties and, in the second year of the campaign, radio ads on targeted FM radio stations in six of the campaign counties. When interpreting the results of the evaluation of *ETC* it is important to note that the campaign did not have the intended effects on motivation and intention to quit smoking among participants exposed to the campaign.

This paper contributes to the current literature by examining awareness of and receptivity to this POS mass media campaign among adult cigarette smokers. We hypothesized that we would find significantly greater awareness of the campaign in treatment participants, than control participants, at all waves of the evaluation and that we would find a larger increase in awareness over time in the treatment group than in the control group. Due to the racial/ethnic disparities in the effects of mass media campaigns that have been identified in previous publications [9, 11, 28] and racial and ethnic differences in receptivity found for previous campaigns [9] (higher receptivity scores for Hispanic Black and Hispanic smokers compared to non-Hispanic white smokers), we hypothesized that we would find differences in receptivity to the campaign by race/ethnicity, and we tested for these differences. The purpose of this analysis was to determine whether cigarette smokers exposed to *ETC* advertisements at the POS noticed the ads and were receptive to them. In addition, the results of this analysis have the potential to inform the use of and content of future mass media campaigns.

## Materials and methods

### Design

To evaluate the campaign, we used a county-level treatment-control longitudinal design with three waves of data collection. Before the start of the evaluation, FCB NY identified 37 counties with a high prevalence of adult smoking and sufficient advertising space for the campaign. Of these counties, 30 were randomly selected for the evaluation; 15 were randomly assigned to be treatment counties, and 15 were assigned to be control counties (S1 Table). To guard against campaign contamination, all control counties were located at least 100 miles from treatment counties. In the US, counties are sub-state territories that are generally (but not always) larger than cities and often use their own government officials to handle local matters.

### Sample

We used an address-based sampling frame to identify 104,000 potentially eligible households in the 30 evaluation counties. Household eligibility criteria were having one or more adults in the household who were 1) between the ages of 25 and 54 years old, 2) currently smoking cigarettes on some days or every day, 3) and not on active military duty. For the first wave of data collection, we sent mail screeners to all identified households and received 19,156 eligible responses. Then, field interviewers visited potentially eligible and nonresponsive households, completed an in-person screener to assess continued eligibility of the household, and, when necessary, randomly selected one eligible adult to participate. Participants provided verbal consent for the in-person screener. If the selected adult agreed, the interviewer administered the wave 1 survey during the same visit.

A total of 3,628 participants (1,712 in treatment counties, 1,916 in control counties) completed the wave 1 survey between June and December 2018 (6–11 months after campaign launch). Trained field interviewers administered the computer-assisted survey via laptops in participants’ homes for all wave 1 interviews. Invitations for participation in waves 2 and 3 were sent by U.S. mail and e-mail, if available. Data collection for wave 2 occurred from March to June 2019 and from August to November 2019 for wave 3. At waves 2 and 3, 42% and 50% of participants, respectively, chose to self-administer the survey online using the link we provided them; all others opted to have an interviewer come to their home and administer the survey in person.

Written informed consent was obtained in all three waves of the survey. Participants received $25 cash for completing the wave 1 survey. Participants who completed the wave 2 and/or 3 surveys online by a specific deadline received a $30 check; all others received a $25 check. The study achieved a 70.8% overall retention rate across the 1.5-year evaluation, with 77.5% retention from wave 1 to 2 and 91.4% retention from wave 2 to 3. The RTI International Institutional Review Board approved all study procedures (approval number 14230).

### Measures

Except for some demographic variables that were not expected to vary over time (e.g., race/ethnicity), measures were captured at all three waves of data collection. All participants received all of the questions described below unless noted otherwise.

#### Outcome variables

*Brand awareness*. To assess brand awareness, participants were shown one randomly selected POS ad in the market at the time and asked, “In the past 3 months, how frequently have you seen or heard the following slogan or theme Every Try Counts?” (see Fig 1 for example ads), with the response options lots of times, a few times, once, or never. Consistent with previous analyses, we dichotomized brand awareness (a few times/lots of times/once versus never) [29, 30].

*Ad awareness*. To assess ad awareness, participants were shown four POS ads (4 print ads at wave 1; 2 print and 2 digital video ads at waves 2 and 3) that were in the market at the time. For all POS ads, participants were asked, “apart from this survey, how frequently have you seen this ad in the past 3 months?” Response options were never, once, a few times, or lots of times. Awareness was coded as reporting having seen one or more of the ads a few times or more often [29, 30]. To guard against order effects, presentation of ads in the survey was randomized. All participants received the ad awareness questions regardless of their response to the brand awareness question.

*Campaign receptivity*. To assess receptivity to campaign messaging, we used a 6-item perceived effectiveness (receptivity) scale that has predicted quit-related beliefs, intentions, and behaviors in evaluations of prior tobacco education campaigns [9, 11]. Participants rated four ads on a 5-point Likert scale from 1 = strongly disagree to 5 = strongly agree for the following statements: 1) This ad is worth remembering; 2) This ad grabbed my attention; 3) This ad is powerful; 4) This ad is informative; 5) This ad is meaningful to me; and 6) This ad is convincing. For each ad, we averaged ratings across the 6 items to create a receptivity score scale from 1 to 5 with high inter-item reliability (average Cronbach alpha = 0.95 across all ads); we also averaged receptivity across all ads at each wave.

#### Exposure variables

*Treatment group*. Participants living in counties with the campaign were considered treatment participants, and participants living in counties without the campaign were considered control participants.

*Demographic characteristics*. To characterize the sample, we included the following wave 1 demographics: age (25–34, 35–44, 45–54), sex (male/female), race/ethnicity (non-Hispanic white, non-Hispanic Black or African American, Hispanic, non-Hispanic other race or multiple races), education (less than high school degree/ no Graduate Equivalency Diploma (GED), high school degree/GED, some college or associate’s degree, 4-year college degree or greater), employment status (employed outside of the home or self-employed, out of work, student/homemaker, or retired/unable to work), and annual household income (<$20,000, $20,000-$49,999, $50,000-$99,999, $100,000 or more).

*Tobacco use*, *purchasing*, *and quit history*. To characterize the sample, we included in this paper wave 1 measures of: number of cigarettes smoked on the most recent day; current use of other tobacco products including e-cigarettes, cigars/cigarillos, hookah, and smokeless tobacco (currently using one or more of these products “every day” or “some days” versus “rarely”, or “not at all”); and, among participants who reported buying their own cigarettes for their own personal use, where they had bought them in the past week (convenience store/ gas station, grocery store, drugstore, mass merchandiser, tobacco shop, or other). Participants also reported whether they had made a quit attempt (not smoking for at least 24 hours because the participant is trying to quit) in the past 3 months, the number of quit attempts in the past 3 months, and the longest period of time they had ever quit smoking completely (in days).

*Additional covariates*. To adjust for potential misattributed campaign awareness and over-reporting bias, we included participants’ self-reported (brand) awareness of the *Tips from Former Smokers*^®^ campaign as a covariate in analyses. Participants were shown a composite image featuring screenshots of two *Tips* broadcast TV ads and asked if they had seen or heard the slogan “Tips from Former Smokers” in the past 3 months. We dichotomized *Tips* awareness (once, a few times, or lots of times versus never).

### Analysis

We used means, frequencies, and percentages to describe outcome variables, predictor variables, and covariates. We used the “svy” command in Stata 17 to adjust all analyses for sampling weights, which accounted for nonresponse bias and any potential unmeasured differences between participants in treatment and control counties at wave 1. To determine if participant characteristics differed by treatment and control group at wave 1, we used the “mean” and “test” commands in Stata to produce Pearson’s F-tests for categorical outcomes and F-tests for continuous variables. We used Pearson’s F-tests to test whether brand and ad awareness differed across the treatment and control groups and whether receptivity differed by race/ ethnicity and treatment group.

To test our hypothesis that we would find a larger difference in the change in awareness over time in the treatment group than in the control group, we used difference in difference (DID) logistic regression models (svy: logistic) to compare the average change in awareness among treatment county participants to the average change in awareness among control county participants. These models enabled us to estimate the treatment effect, or change, in the treatment group attributable to the intervention by regressing wave 3 values of the outcome variables (brand awareness and ad awareness, in separate models) on wave 1 values for the same variables, consistent with the methods used to evaluate previous media campaigns [29, 30]. The DID models included a treatment x wave interaction term to assess whether changes in awareness from wave 1 to wave 3 were greater in treatment counties than control counties; we dropped this term from the model if it was not significant. For all models, we included treatment group, wave, and, to adjust for pre-existing treatment-control differences unrelated to the campaign, we included wave 1 covariates that differed significantly by treatment group and provided the best model fit based on the Akaike Information Criterion and Bayesian Information Criterion.

All data were weighted to account for differences in the demographic characteristics of the treatment and control groups at wave 1. Waves 1 and 2 are weighted by wave 1 weights due to a lack of changes in the sample, which can be caused by attrition, between waves. At wave 3, we created new weights to account for changes in the sample between waves 2 and 3. Wave 3 values are weighted by wave 3 weights.

## Results

### Descriptive and bivariate results

Demographics and Tobacco Use Variables. Overall, the majority of the sample was non-Hispanic White (56.0%) and currently employed (65.6%), with a slightly greater percentage of women (53.6%) than men (Table 1). Over 75% of the sample reported an annual household income of less than $50,000 per year, and approximately 57% had not completed education beyond high school. On average, the sample reported smoking more than half a pack of cigarettes a day, and the vast majority (92.6%) bought their cigarettes at convenience stores or gas stations. Over a quarter of the sample had made at least one quit attempt in the past 3 months.

**Table 1:** Wave 1 sample characteristics^1^ in the 15 treatment and 15 control counties in the *Every Try Counts* evaluation (n = 3,628).

	Full Sample N = 3,628	Counties		Treatment/ Control Group Difference		
		Treatment N = 1,712	Control N = 1,916			
	Percent/Mean (n/ SE)	Percent/Mean (n/ SE)	Percent/Mean (n/ SE)	p-value	F-statistic	df
**Age (Years)**				0.017	4.08	3,627
25–34	36.2% (1,184)	35.0% (537)	38.2% (647)			
35–44	29.6% (1,141)	27.9% (522)	32.6% (619)			
45–54	34.3% (1,303)	37.0% (653)	29.1% (650)			
**Sex**						
Female	53.2% (1,953)	53.6% (948)	52.4% (1,005)	0.695	0.15	3,627
Male	46.8% (1,675)	46.4% (764)	47.6% (911)			
**Race/Ethnicity**				0.001	5.51	3,626
Non-Hispanic white	49.1% (2,032)	48.8% (920)	49.6% (1,112)			
Non-Hispanic Black	31.9% (944)	35.0% (524)	26.1% (420)			
Hispanic	10.2% (313)	8.7% (133)	13.0% (180)			
Non-Hispanic other/ multiple races	8.8% (338)	7.5% (134)	11.3% (204)			
**Education**				0.774	0.37	3,625
Less than high school degree	18.5% (705)	18.4% (349)	18.7% (356)			
High school degree or GED	39.9% (1,369)	40.4% (649)	38.9% (720)			
Some college or associate’s degree	31.1% (1,231)	30.2% (553)	32.6% (678)			
4-year college degree or greater	10.6% (321)	11.0% (160)	9.7% (161)			
**Employment Status**				0.042	2.75	3,621
Employed	65.7% (2,379)	65.0% (1,114)	66.9% (1,265)			
Out of work	11.3% (372)	11.0% (173)	11.9% (199)			
Student/ homemaker	7.4% (307)	6.4% (141)	9.0% (166)			
Retired/ unable to work	15.7% (564)	17.6% (284)	12.1% (280)			
**Annual Household Income**				0.730	0.47	3,218
Less than $20,000	39.0% (1,390)	38.5% (664)	40.1% (726)			
$20,000 to $49,999	35.2% (1,292)	34.8% (616)	35.9% (676)			
$50,000 to $99,999	19.% (653)	20.7% (305)	17.9% (348)			
$100,000+	6.0% (197)	6.0% (84)	6.0% (113)			
**Want to Quit Smoking Cigarettes Somewhat or a Lot**	73.3% (2,070)	73.9% (1,016)	72.2% (1,054)	0.590	0.29	2,859
**Intention to Quit Smoking in the Next 30 Days**	10.8% (404)	10.4% (195)	11.5% (209)	0.484	0.49	3,627
**Have Set a Firm Quit Date**	14.1% (288)	14.8% (149)	12.8% (139)	0.446	0.58	2,177
**Number of Cigarettes Smoked on the Most Recent Day**	13.11 (0.48)	13.59 (0.71)	12.19 (0.35)	0.707	0.78	3,563
**Current Other Tobacco Use** ^2^	26.5% (965)	31.2% (472)	29.3% (493)	0.537	0.38	3,627
**Usually Buy Own Cigarettes**	94.0% (3,445)	93.5% (1,638)	94.8% (1,807)	0.391	0.73	3,625
**Cigarette Purchasing Source (Past Week)** ^3^						
Convenience store or gas station	92.6% (2,945)	92.3% (1,416)	93.0% (1,529)	0.620	0.25	3,236
Grocery store	28.4% (778)	30.6% (383)	24.3% (395)	0.042	4.14	3,225
Drugstore	15.8% (323)	18.1% (172)	11.6% (151)	0.011	6.53	3,215
Mass merchandizer	13.9% (435)	12.6% (200)	16.4% (235)	0.124	2.36	3,218
Tobacco shop	26.6% (966)	24.5% (387)	30.6% (579)	0.029	4.75	3,223
Other retailer	12.6% (295)	13.5% (157)	10.8% (138)	0.217	1.52	3,121
**Made Quit Attempt in Past 3 Months** ^4^	28.3% (925)	28.7% (443)	27.5% (482)	0.688	0.16	3,625
**Number of Quit Attempts, Past 3 Months**	1.78 (0.23)	1.73 (0.29)	1.89 (0.36)	0.325	1.12	3,586
**Longest Period Abstinent from Cigarettes (Days)**	424.64 (41.17)	442.68 (34.92)	390.93 (41.29)	0.002	1.73	2,862
**Brand Awareness of *Tips*** ^5^	70.4% (2,569)	71.1% (1,258)	69.2% (1,311)	0.509	0.44	3,626
**Brand awareness for *Every Try Counts*** ^6^	44.6% (1,496)	48.2% (805)	37.8% (691)	<0.001	12.40	3,627
**Ad Awareness for *Every Try Counts*** ^7^	29.7% (914)	33.1% (507)	23.2% (407)	<0.001	13.40	3,627

Note: We used Pearson’s F-tests to test for differences in categorical outcomes and F-tests for differences in continuous variables. We used the svy command to weight all analyses by sampling weights.

^1^ All estimates in table are weighted for nonresponse bias and to calibrate the treatment and control group to each other to ensure that the demographics of the two groups were as similar as possible; weights were updated when needed due to changes in the sample over time (i.e., differential loss to follow-up).

^2^ Some days or everyday use of electronic vapor products, hookah, smokeless tobacco, and/or cigar/cigarillo/little cigars

^3^ Only participants who reported purchasing their own cigarettes answered this question.

^4^ Quit attempts were defined as stopping smoking for 24 hours or more because the participant was trying to quit smoking.

^5^ Reported having seen or heard the slogan “Tips from Former Smokers” in the past 3 months once or more times (as opposed to never).

^6^ Reported having seen or heard the slogan “Every Try Counts” at least once in the past 3 months (as opposed to never).

^7^ Reported seeing one or more Every Try Counts ads a few times or more often in the past 3 months (as opposed to never or once).

Because randomization was conducted at the county level (as opposed to the individual level), a few significant differences existed in the characteristics of the treatment groups at wave 1 (Table 1). Participants in treatment counties were older (p = 0.017) and more likely to be retired or disabled (p = 0.042). We also found a larger proportion of racial and ethnic minority participants in treatment counties than in control counties (p = 0.001). To adjust for pre-existing differences between treatment and controls, we included age group, employment status, and race/ethnicity in regression models. We also included *Tips* awareness in all models because it improved model fit.

#### Brand and ad awareness

For brand awareness, 48.2% of participants in treatment counties reported having seen or heard the slogan “Every Try Counts” at least once in the past 3 months at wave 1 compared to 37.8% of those in control counties (Table 1). Brand awareness increased to 55.5% by wave 2 and remained at 54.0% at wave 3 among treatment county participants, compared to 37.9% at wave 2 and 40.6% at wave 3 among control county participants.

For ad awareness, at wave 1, 33.1% of participants in treatment counties reported having seen at least one POS ad a few times or more often in the past 3 months, compared to 23.2% of controls. Ad awareness increased to 41.6% by wave 2 and 53.3% by wave 3 among treatment participants, compared to 31.4% and 36.1%, respectively, for waves 2 and 3 among control participants.

Brand awareness was significantly higher among participants in treatment than control counties at all waves (all p < 0.001; wave 1: F = 12.40, df = 3,622). Similarly, ad awareness was significantly higher among participants in treatment than control counties at all waves (all p < 0.001; wave 1: F = 13.40, df = 3,627).

#### Receptivity

Receptivity scores for the treatment group, averaged across all ads, were 3.27 (95%CI: 3.20–3.24) at wave 1, 3.19 (95%CI: 3.12–3.27) at wave 2, and 3.26 (95%CI: 3.19–3.34) at wave 3 (Table 2). Receptivity scores for individual print ads ranged from 3.24 (95%CI: 3.15–3.33) for “You didn’t fail at quitting” and “Keep your head up” (95%CI: 3.14–3.33) at wave 1 to 3.34 (95%CI: 3.25–3.43) for “Never quit quitting” at wave 3. Receptivity for individual digital ads ranged from 3.00 (95%CI: 2.90–3.10) for “Guitar” at wave 2 to 3.27 (95%CI: 3.18–3.36) for “Baseball” at wave 3.

**Table 2:** Campaign receptivity for wave 1–3 *Every Try Counts* ads among treatment county participants—Full sample and by race/ethnicity.

	Mean [95%CI]					p-value	F-stat	df
	Overall	Non-Hispanic White	Non-Hispanic Black	Hispanic	Non-Hispanic Other/Multiple races			
**Treatment Group**								
**Wave 1 Ads**								
All ads	3.27 [3.20–3.24]	3.11 [3.01–3.22]	3.49 [3.38–3.59]	3.37 [3.17–3.57]	3.20 [2.98–3.42]	< 0.001	8.85	1,305
You didn’t fail at quitting	3.24 [3.15–3.33]	3.08 [2.94–3.21]	3.42 [3.30–3.55]	3.44 [3.17–3.71]	3.23 [2.98–3.49]	<0.001	4.98	1,261
If at first you don’t succeed	3.28 [3.19–3.37]	3.13 [2.99–3.27]	3.48 [3.34–3.63]	3.35 [3.11–3.58]	3.22 [3.00–3.44]	<0.001	4.14	1,305
Every time you put out a cigarette	3.28 [3.25–3.40]	3.19 [3.08–3.30]	3.54 [3.41–3.68]	3.42 [3.24–3.60]	3.21 [2.89–3.53]	<0.001	4.58	1,283
Keep your head up	3.24 [3.14–3.33]	3.05 [2.91–3.20]	3.54 [3.41–3.68]	3.28 [3.01–3.55]	3.13 [2.84–3.41]	<0.001	8.16	1,267
**Wave 2 Ads**								
All ads	3.19 [3.12–3.27]	3.04 [2.94–3.13]	3.43 [3.31–3.55]	3.26 [3.01–3.50]	2.98 [2.77–3.20]	< 0.001	9.56	1,308
Learn something	3.32 [3.25–3.40]	3.11 [3.02–3.21]	3.60 [3.47–3.72]	3.43 [3.16–3.71]	3.23 [3.02–3.43]	<0.001	12.00	1,308
Never quit quitting	3.30 [3.22–3.38]	3.15 [3.05–3.24]	3.50 [3.35–3.65]	3.43 [3.17–3.68]	3.19 [2.95–3.44]	<0.001	5.85	1,308
Guitar (digital video)	3.00 [2.90–3.10]	2.90 [2.75–3.04]	3.22 [3.07–3.38]	2.97 [2.63–3.31]	2.58 [2.19–2.97]	0.003	4.84	1,306
Baseball (digital video)	3.14 [3.05–3.24]	2.98 [2.85–3.11]	3.38 [3.24–3.53]	3.19 [2.85–3.52]	2.93 [2.57–3.30]	<0.001	5.90	1,307
**Wave 3 Ads**								
All ads	3.26 [3.19–3.34]	3.17 [3.09–3.26]	3.37 [3.22–3.52]	3.39 [3.10–3.68]	3.13 [2.93–3.33]	<0.001	2.39	1,193
Learn something	3.30 [3.21–3.39]	3.16 [3.06–3.25]	3.45 [3.27–3.62]	3.41 [3.12–3.70]	3.28 [3.09–3.46]	<0.009	3.27	1,193
Never quit quitting	3.34 [3.25–3.43]	3.21 [3.10–3.32]	3.49 [3.33–3.66]	3.43 [3.10–3.75]	3.20 [2.96–3.44]	0.001	2.98	1,192
Guitar (digital video)	3.15 [3.06–3.25]	3.13 [3.01–3.25]	3.19 [3.01–3.36]	3.31 [3.01–3.62]	2.89 [2.59–3.19]	0.010	1.39	1,192
Baseball (digital video)	3.27 [3.18–3.36]	3.19 [3.07–3.31]	3.36 [3.18–3.53]	3.40 [3.07–3.72]	3.15 [2.90–3.39]	0.289	1.30	1,192
**Control Group**								
**Wave 1 Ads**								
All ads	3.17 [3.11–3.24]	3.10 [3.02–3.19]	3.31 [3.20–3.42]	3.14 [2.86–3.43]	3.20 [3.01–3.39]	0.029	3.00	1,420
You didn’t fail at quitting	3.15 [3.07–3.23]	3.06 [2.95–3.17]	3.27 [3.14–3.39]	3.22 [2.90–3.53]	3.23 [3.00–3.47]	0.090	2.17	1,417
If at first you don’t succeed	3.16 [3.07–3.24]	3.13 [3.02–3.24]	3.30 [3.15–3.44]	2.82 [2.45–3.20]	3.25 [2.99–3.51]	0.073	2.33	1,419
Every time you put out a cigarette	3.18 [3.10–3.26]	3.09 [2.99–3.20]	3.41 [3.26–3.55]	3.17 [2.85–3.48]	3.11 [2.91–3.31]	0.006	4.16	1,416
Keep your head up	3.20 [3.11–3.29]	3.13 [3.02–3.23]	3.28 [3.12–3.43]	3.29 [2.92–3.66]	3.22 [2.99–3.44]	0.424	0.93	1,418
**Wave 2 Ads**								
All ads	3.11 [3.04–3.18]	3.01 [2.93–3.09]	3.22 [3.09–3.34]	3.16 [2.85–3.48]	3.22 [3.02–3.42]	0.023	3.19	1,420
Learn something	3.20 [3.12–3.27]	3.08 [2.98–3.17]	3.31 [3.17–3.45]	3.37 [3.06–3.67]	3.26 [3.06–3.46]	0.015	3.50	1,417
Never quit quitting	3.16 [3.08–3.25]	3.08 [2.99–3.17]	3.30 [3.16–3.45]	3.13 [2.75–3.51]	3.25 [3.03–3.46]	0.062	2.45	1,419
Guitar (digital video)	2.99 [2.90–3.07]	2.91 [2.80–3.03]	3.07 [2.93–3.22]	3.03 [2.70–3.36]	3.07 [2.84–3.30]	0.316	1.18	1,416
Baseball (digital video)	3.08 [2.99–3.18]	2.98 [2.86–3.09]	3.16 [3.02–3.31]	3.13 [2.73–3.53]	3.30 [3.05–3.55]	0.059	2.48	1,418
**Wave 3 Ads**								
All ads	3.14 [3.06–3.23]	3.08 [2.98–3.19]	3.19 [3.03–3.35]	3.18 [2.88–3.49]	3.23 [2.99–3.47]	0.574	0.67	1,300
Learn something	3.19 [3.11–3.27]	3.05 [2.94–3.17]	3.38 [3.23–3.53]	3.21 [2.93–3.48]	3.32 [3.09–3.55]	0.005	4.33	1,299
Never quit quitting	3.19 [3.10–3.28]	3.07 [2.95–3.20]	3.35 [3.19–3.52]	3.16 [2.83–3.49]	3.32 [3.06–3.59]	0.041	2.76	1,299
Guitar (digital video)	3.03 [2.91–3.14]	3.07 [2.95–3.20]	2.93 [2.73–3.13]	2.97 [2.37–3.57]	3.09 [2.81–3.37]	0.666	0.52	1,300
Baseball (digital video)	3.17 [3.07–3.26]	3.14 [3.02–3.25]	3.09 [2.88–3.30]	3.40 [3.11–3.69]	3.19 [2.91–3.46]	0.371	1.05	1,299

Note: We used F-tests to test for differences in continuous variables. We used the svy command to weight all analyses by sampling weights. CI = confidence interval

Receptivity scores are the mean of ratings (on a 5-point Likert scale from 1 = strongly disagree to 5 = strongly agree) for the following items: 1) This ad is worth remembering; 2) This ad grabbed my attention; 3) This ad is powerful; 4) This ad is informative; 5) This ad is meaningful to me; and 6) This ad is convincing.

Receptivity scores for the control group, averaged across all ads, were 3.17 (95%CI: 3.11–3.24) at wave 1, 3.11 (95%CI: 3.04–3.18) at wave 2, and 3.14 (95%CI: 3.06–3.23) at wave 3 (Table 2). Receptivity scores for individual print ads ranged from 3.15 (95%CI: 3.07–3.23) for “You didn’t fail at quitting” at wave 1 to 3.20 (95%CI: 3.11–3.29) for “Keep your head up” at wave 1 and 3.20 (95%CI: 3.12–3.27) for “Learn something” at wave 2. Receptivity for individual digital ads ranged from 2.99 (95%CI: 2.90–3.07) for “Guitar” at wave 2 to 3.17 (95%CI: 3.07–3.26) for “Baseball” at wave 3. For both treatment and control groups, none of the differences in receptivity to ads at each wave were significant at the p < 0.05 level.

We found a few significant differences in campaign receptivity for comparisons by treatment group. At all three waves, receptivity averaged across all ads was higher in the treatment than control group (wave 1: p = 0.010, wave 2: p = 0.048, wave 3: p = 0.010). There was also only one significant difference between the treatment and control group for individual ads. Receptivity for “If at first you don’t succeed” was higher in the treatment than control group (p = 0.041).

We also compared receptivity by race and ethnicity within the treatment and control groups. In the treatment group, we found significantly higher receptivity scores for non-Hispanic Black and Hispanic participants (p < 0.001 for trend), compared to non-Hispanic white participants, for receptivity averaged across all ads and for many individual ads (all p < 0.010, see Table 2 for exact p-values), with the exception of the digital video ad “Baseball” at wave 3 (p = 0.289; Table 2).

As was the case with the treatment group, in the control group, we found higher receptivity scores for non-Hispanic Black and non-Hispanic other race participants, compared to all other racial/ethnic groups, for scores averaged across all ads at waves 1 (p = 0.029 for trend) and 2 (p = 0.023 for trend). We also found some differences for individual ads. For “Every time you put out a cigarette,” receptivity was significantly higher for non-Hispanic Black participants than for all other racial/ethnic groups (p = 0.006 for trend). Scores were lower for non-Hispanic white participants for the “Learn something” ad compared to all other racial/ethnic groups at Waves 2 (p = 0.015 for trend) and 3 (p = 0.005 for trend). In addition, at wave 3, scores for “Never quit” were higher for non-Hispanic Black and non-Hispanic other race participants (p = 0.041).

### Regression models

Initial DID models included an interaction term for treatment group by time. The interaction term was not significant for either brand awareness (p = 0.688) or ad awareness (p = 0.339); therefore, we dropped this term from both models (Table 3). The models presented below do not include the interaction term. Although we tested all of the exposure variables included in the measures section for inclusion in DID models for brand and ad awareness, only age, race/ethnicity, employment status, and *Tips* awareness improved model fit and were included in the models.

**Table 3:** Logistic regression models for *Every Try Counts* brand and ad awareness.

Predictors	Awareness			
	Brand^1^		Ad^2^	
	OR	CI	OR	CI
**Treatment**				
Control	Ref	Ref	Ref	Ref
Treatment	1.53	[1.26,1.86]	1.74	[1.39,2.16]
**Wave**				
Wave 1	Ref	Ref	Ref	Ref
Wave 3	1.25	[1.02,1.53]	2.21	[1.84,2.65]
**Age Group**				
25–34	Ref	Ref	Ref	Ref
35–44	0.89	[0.68,1.16]	1.19	[0.89,1.61]
45–54	0.81	[0.63,1.04]	1.01	[0.80,1.49]
**Race/Ethnicity**				
Non-Hispanic white	Ref	Ref	Ref	Ref
Non-Hispanic Black	1.70	[1.34,2.16]	1.76	[1.33,2.33]
Hispanic	1.20	[0.82,1.75]	1.68	[1.21,2.34]
Non-Hispanic other/ multiple races	1.19	[0.87,1.62]	1.43	[0.94,2.16]
**Employment**				
Employed	Ref	Ref	Ref	Ref
Out of work	0.91	[0.68,1.21]	0.88	[0.60,1.27]
Student/ homemaker	0.99	[0.67,1.43]	1.35	[0.96,1.89]
Retired/ unable to work	1.10	[0.79,1.53]	1.34	[0.87,2.06]
***Tips* Brand Awareness** ^3^	2.66	[2.12,3.35]	1.78	[1.37,2.31]
Model constant	0.13	[0.09, 0.17]	0.27	[0.21, 0.36]

Note: We used the svy command to weight all analyses by sampling weights. OR = odds ratio, CI = 95% confidence interval.

^1^ Reported seeing the ETC slogan/theme once or more often in the past 3 months, model df = 3,619, F = 12.16, p<0.001.

^2^ Reported seeing the ETC ads a few times or more often in the past 3 months, model df = 3,616, F = 11.86, p< 0.001.

^3^ Reported having seen or heard the slogan “Tips from Former Smokers” in the past 3 months once or more times (as opposed to never).

#### Adjusted model for brand awareness

In the final adjusted model, awareness was significantly higher at wave 3 (OR = 1.25, 95%CI: 1.02–1.53) than at wave 1. Treatment county participants had greater odds of reporting awareness of the *ETC* brand than control county participants (OR = 1.53, 95%CI: 1.26–1.86). Non-Hispanic Black participants had higher awareness of the *ETC* brand than non-Hispanic white participants (OR = 1.70, 95%CI: 1.34–2.16), as did participants reporting awareness of the *Tips* campaign (OR = 2.66, 95%CI: 2.12–3.35).

#### Adjusted model for ad awareness

In the final adjusted model, ad awareness at wave 3 was significantly higher than awareness at wave 1 (OR = 1.25, 95%CI: 1.02–1.53). The treatment group had greater odds of reporting ad awareness (OR = 1.74, 95%CI: 1.393–2.16) than the control group. Both non-Hispanic Black (OR = 1.76, 95% CI: 1.33–2.33) and Hispanic (OR = 1.68, 95%CI: 1.21–2.34) participants reported greater ad awareness than non-Hispanic white participants. Participants who reported awareness of *Tips* (OR = 1.78, 95%CI: 1.37–2.31) also had higher awareness of *ETC* ads.

## Discussion

This paper presents results from FDA’s *ETC* tobacco education campaign, which, to our knowledge, was the first multi-county POS campaign in the U.S. Brand and ad awareness were higher in the treatment group than the control group at all waves, and receptivity levels were consistent with those of other (non-point-of-sale) tobacco control campaign evaluations [9, 29, 30]. Receptivity differed significantly by race and ethnicity. We did not find a significant difference in the change in ad awareness over time by treatment group.

As expected, brand and ad awareness were significantly higher in treatment counties than in control counties, with over half of treatment participants reporting having seen the *ETC* brand and POS ads 20 months into the campaign. Although campaign awareness was lower than the CDC’s recommended goal of reaching 75% to 85% of the target audience to achieve sufficient exposure [25], it is important to note that CDC’s benchmark is based on evidence from primarily broadcast media campaigns. Given the overwhelming array of distracting signage in typical convenience stores [20, 24] and that consumers spend less than 5 minutes, on average, inside these stores [31], awareness levels are likely to be lower for POS campaigns than for other types of campaigns.

Receptivity scores for ads were higher in the treatment than control group at all waves for scores averaged across all ads. This finding is consistent with the existing literature. Zhao et al. [32] found a significant positive relationship between exposure to *The Real Cost* campaign and PE. They also found a significant positive relationship between awareness of the *truth*^*®*^ campaign and PE [32]. This finding is also consistent with previous studies that have shown higher receptivity to tobacco advertising with repeated exposure to these ads [33, 34].

In regression models, brand and ad awareness were higher among Hispanic and non-Hispanic Black smokers after adjusting for treatment group and other covariates. Previous evaluations of mass media tobacco education campaigns, such as the 2009 smoking cessation campaign “Become an EX” (or “EX”), have found greater awareness among non-Hispanic Black smokers [10]. Although, like *ETC*, the EX campaign used a supportive approach to cessation messaging, it was not a POS campaign. Also relevant for this finding is that, in an analysis using data from the Population Assessment of Tobacco and Health study, Groom et al. [3] found that non-Hispanic Black participants were more likely to report that they had noticed in-store tobacco ads or promotions than non-Hispanic white participants. It is unclear, however, whether this finding can be applied to tobacco education ads at the POS.

In bivariate analyses, we found higher receptivity among non-Hispanic Black and Hispanic smokers in the treatment and control groups. This finding is consistent with evaluations of national broadcast media campaigns [9]. Non-Hispanic Black and Hispanic smokers were significantly more receptive to 2014 *Tips From Former Smokers* ads than non-Hispanic white smokers [9]. *Tips* is not a POS campaign, however, and the study was not able provide any explanations for these differences. Additional research is needed to determine the explanation for higher reported campaign awareness and receptivity among racial and ethnic minority populations.

For the control group, but not the treatment group, we noted significant variation in receptivity to individual ads by race and ethnicity. One explanation for this finding is differences in reactions to ads based on the race/ethnicity of the respondent and the race/ethnicity of the actor or spokesperson in the ad [35, 36]. Some of our results for the control group generally support this explanation. At wave 1, receptivity was significantly higher for non-Hispanic Black participants for “Every time you put out a cigarette,” which was the only ad at wave 1 that featured a Black actor. At waves 2 and 3, receptivity for “Learn something” was higher for non-Hispanic Black, Hispanic, and non-Hispanic other race participants; this ad featured an African American woman. At wave 3, non-Hispanic Black and Hispanic participants rated the “Never quit” ad higher, and the actress was of mixed race. However, we did not find differences by race and ethnicity based on actor and respondent race for either of the digital ads shown at waves 2 and 3.

One interesting finding is that 23% to 36% of smokers in the control counties reported that they had seen the *ETC* ads. Given the place-based design of the campaign and efforts to prevent campaign spillover into control counties, it is unlikely that smokers in control counties were exposed to *ETC*. This finding of false ad recognition is not unique to our study; Coady and colleagues found a similar pattern with 30% of smokers reporting having seen the NYC Board of Health’s graphic warning signs in grocery stores and pharmacies prior to actual installation of the signs [19]. Several factors may have contributed to incorrectly reported campaign awareness by smokers in control counties. First, our study relied on an aided recall method in which participants were shown examples of campaign ads in the survey prior to answering questions about awareness, which could have led to over-reporting, recall bias, and increases in awareness in the control group over time. However, it is difficult to use alternative methods, such as confirmed awareness techniques, in online surveys. Approximately half of participants completed the survey online at waves 2 and 3. Second, smokers in control counties may have confused exposure to other local, state, and national smoking cessation campaigns with purported exposure to *ETC* ads, as suggested by the significance of *Tips* awareness in regression models.

Third, although supportive messaging may be effective for smoking cessation, more salient graphic and emotional messages may be necessary to garner attention at the POS and for smokers to accurately recall whether they have seen these messages [20, 24, 37, 38]. However, retailers are unlikely to consent to having emotional and graphic ads placed at the POS for fear of the impact on tobacco sales.

We did not find significant differences in changes in awareness over time between the treatment and control groups. Although this finding is not completely surprising given that the campaign had been in place several months before data collection began (i.e., there was no baseline data collection), we anticipated finding a greater increase in awareness among members of the treatment group. This finding is likely due to the same factors (discussed above) that resulted in higher-than-expected levels of awareness in the control group.

### Limitations

One limitation of the study was the reliance on self-report data for awareness. Another limitation is generalizability. The evaluation only included 30 U.S. counties; therefore, the results of this analysis may not be generalizable beyond these 30 counties. The results are most likely to represent smokers living in counties with a high smoking prevalence and sufficient POS retail space to support a POS-based campaign. Although limitations to generalizability remain, our randomized treatment-control design should have somewhat reduced the influence of county-level characteristics on our results. Another limitation is the short time period between waves 2 and 3, which may have affected our ability to see changes in awareness in the treatment group during this time.

### Conclusion

Tobacco control advocates and policy makers have pointed to POS education campaigns as a strategy for countering tobacco industry price promotions and other marketing tactics [39]. Yet, prior to *ETC*, only one U.S. city had implemented and evaluated prevention and cessation messaging at the POS. The evaluation of the *ETC* campaign found that supportive messaging delivered at the POS attracted some attention from cigarette smokers and that smokers were receptive to these ads; receptivity varied by race/ethnicity. However, the difference in ad awareness between the treatment and control groups was smaller than expected. Augmenting a POS campaign with complementary messaging on mass reach media channels (e.g., broadcast television) may be needed to ensure sufficient levels of awareness.

## Supporting information

S1 File Additional information on Gross Ratings Points (GRPs) for *Every Try Counts*. This document provides additional information on the approach to measuring GRPs for this campaign and the GRP levels achieved by the *Every Try Counts* campaign.
(PDF)

S1 Table Counties included in the *Every Try Counts* evaluation. This table shows the treatment and control counties included in the evaluation of the *Every Try Counts* campaign.
(PDF)
