# Supplementary material for: Awareness of and receptivity to FDA’s point-of-sale tobacco public education campaign
Source: PLoS One. 2023 Jul 13;18(7):e0288462. doi: 10.1371/journal.pone.0288462 (PMC10343043; doi:10.1371/journal.pone.0288462)
Supplement: S1 File — This document provides additional information on the approach to measuring GRPs for this campaign and the GRP levels achieved by the Every Try Counts campaign. (PDF) [file pone.0288462.s001.pdf]

To measure objective campaign exposure, we used a modified version of quarterly gross rating points (GRPs) based on a formula developed by FCB New York. The formula included 1) vendor-supplied average monthly store traffic and number of tactics displayed (e.g., posters, gas pump toppers) and 2) county prevalence of smoking, percentage of smokers who visit convenience stores, and visibility of each tactic to calculate the total number of smoker impressions (i.e., number of times ads are seen by adult smokers). The number of smoker impressions were then divided by the total population of adults 18 and older in each county and multiplied by 100. We only include wave 1 GRPs in our analysis because GRPs remained consistent throughout the evaluation.

In June 2018, 6 months after campaign launch, quarterly GRPs ranged from 1,024 to 4,534 across the 15 treatment counties. Every county exceeded the CDC-recommended dose of 800 GRPs per quarter. Quarterly GRPs remained stable throughout the 2-year campaign (< 3% variation over time).
