# Supplementary material for: Awareness of and receptivity to FDA’s point-of-sale tobacco public education campaign
Source: PLoS One. 2023 Jul 13;18(7):e0288462. doi: 10.1371/journal.pone.0288462 (PMC10343043; doi:10.1371/journal.pone.0288462)
Supplement: S1 Table — This table shows the treatment and control counties included in the evaluation of the Every Try Counts campaign. (PDF) [file pone.0288462.s002.pdf]

**Treatment Counties**	**Control Counties**
Bristol County, MA	Wyandotte County, KS
Philadelphia County, PA	Monroe County, PA
Washington County, MD	Baltimore City, MD
Citrus County, FL	Volusia County, FL
Hillsborough County, FL	Norfolk City, VA
Coweta County, GA	Grayson County, TX
Lowndes County, GA	Brazos County, TX
Marshall County, AL	St. Clair County, AL
Wilson County, TN	Johnston County, NC
Lake County, IL	St. Joseph County, IN
Trumbull County, OH	Floyd County, KY
Wayne County, MI	Oklahoma County, OK
St Louis City, MO	Muskogee County, OK
Milwaukee County, WI	Anoka County, MN
Stanislaus County, CA	Butte County, CA
